# Invasive tracheobronchial aspergillosis developed during radioimmunotherapy for malignant lymphoma

**DOI:** 10.1002/ccr3.1453

**Published:** 2018-03-04

**Authors:** Shuku Sato, Yotaro Tamai, Hideyasu Sugimoto, Shinji Watanabe, Tomohiro Kumagae, Emiko Kannbe, Eri Tanaka

**Affiliations:** ^1^ Division of Hematology Shonan Kamakura General Hospital Kanagawa Japan; ^2^ Division of Respiratory Medicine Shonan Kamakura General Hospital Kanagawa Japan; ^3^ Division of General Medicine Shonan Kamakura General Hospital Kanagawa Japan

**Keywords:** Immunodeficiency, invasive tracheobronchial aspergillosis, malignant lymphoma, radioimmunotherapy

## Abstract

Invasive pulmonary aspergillosis (IPA) often occurs during the treatment of malignant lymphoma. However, invasive tracheobronchial aspergillosis (ITBA) is a rare form of IPA. Particularly, due to the decrease in immunity associated with chemotherapy, it is difficult to diagnose ITBA only by CT imaging and serological findings. Pathologic diagnosis by bronchoscopy is important.

## Introduction

A 69‐year‐old female presented with fever and dyspnea 15 days after commencing radioimmunotherapy (RIT) for malignant lymphoma. She was diagnosed with invasive tracheobronchial aspergillosis (ITBA). We report a rare case of ITBA acquired during lymphoma treatment, particularly RIT, successfully managed using low‐dose PSL and antifungal therapy.

Infection with *Aspergillus* species occurs via the inhalation of spores that cause a variety of clinical syndromes, ranging from aspergilloma to severe fulminant invasive pulmonary aspergillosis (IPA) 1. IPA is a rare but life‐threatening infection among immunocompromised patients, particularly patients with prolonged neutropenia and inherited immunodeficiency, as well as patients receiving high‐dose corticosteroids or immunosuppressive therapies and those who have undergone allogeneic hematopoietic stem cell transplantation 2. Leukemia and lymphoma are the two most common hematological malignancies predisposing patients to IPA 3. However, invasive tracheobronchial aspergillosis (ITBA), in which the infection is entirely limited or predominantly confined to tracheobronchial lesions, is a relatively rare form of IPA 4. Although ITBA may often develop after lung transplantation 5, it is rarely developed during the treatment of malignant lymphoma.

Radioimmunotherapy (RIT) with yttrium‐90 ibritumomab tiuxetan is one of the treatment options for relapsed and refractory low‐grade non‐Hodgkin lymphoma 6.

Radioimmunotherapy resulted in the depletion of peripheral blood B cells for 6–9 months. This treatment is well tolerated, even by elderly or frail patients, due to its low toxicity 7.

We present a rare case of ITBA during RIT for malignant lymphoma. The patient presented with respiratory failure owing to *Aspergillus* mucoid impaction, which was successfully managed with antifungal therapy. Some clinical features of this case were uncommon for ITBA, owing to RIT and prior salvage chemotherapies.

## Case Report

**Figure 1:**
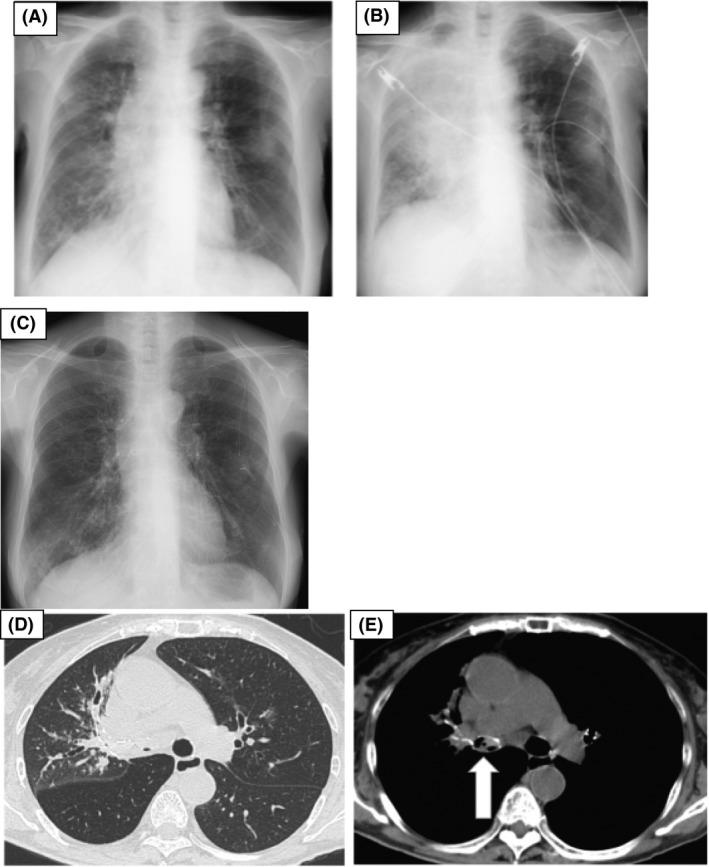
(A) Chest X‐ray on admission showed a mediastinal shift to the right for atelectasis, a reticulonodular pattern on the right lower lobe, and nodular density on the left middle lobe. (B) Ten hours after admission, permeability of the right lung was reduced due to massive atelectasis on the right lung field. (C) Seven days after admission, the atelectasis improved. (D, E) Computed tomography showed mucoid impaction and thickened bronchial walls in the right main bronchus, bronchodilation in the right middle lobar bronchus (arrow), and a centrilobular reticulonodular shadow in the distal lung field.

The patient was a 69‐year‐old woman with a right cervical lymphadenopathy and face edema associated with superior vena cava syndrome caused by a mediastinal tumor. The patient had initially been diagnosed with diffuse large B‐cell lymphoma, but the diagnosis was corrected to mantle‐cell lymphoma after the patient received four cycles of R‐CHOP (rituximab, cyclophosphamide, doxorubicin, vincristine, and prednisolone) chemotherapy, with a partial response. A partial response was also observed following salvage chemotherapy, including two courses of R‐Hyper CVAD therapy (rituximab, cyclophosphamide, vincristine, doxorubicin, and dexamethasone) and two courses of R‐MA therapy (rituximab, methotrexate, and cytarabine). Although other salvage chemotherapy treatments were proposed, the patient received RIT therapy mainly because of its low toxicity. However, she developed a minor cough and sputum overproduction, and at 15 days after RIT therapy, the patient was admitted to the emergency department of our hospital complaining of dyspnea. The patient's body mass index was 18.0 kg/m^2^ (weight, 40.6 kg; height, 150 cm). Physiological examination revealed the following: blood pressure of 125/75 mmHg, pulse rate of 123 beats per minute, body temperature of 37.7°C, respiratory rate of 36 breaths per minute, and oxygen saturation of 96%. The patient received 5 L/min oxygen supplementation via a mask. Her breathing sounds were weak, and wheezing was audible at the end of expiration in both lungs. The patient's white blood cell count was 1.1 × 10^9^/L, with 58.4% neutrophils, 8.7% lymphocytes, 9.0% monocytes, 23.3% eosinophils, and 0.6% basophils. C‐reactive protein and lactate dehydrogenase levels were elevated at 27.45 mg/dL (normal range, 0–0.5 mg/dL) and 285 U/L (normal range, 110–229 U/L), respectively, and the *β*‐D‐glucan level was 26.5 pg/mL (normal range, 0–20 pg/mL). CD4 and CD8 levels were 98.1/*μ*L and 95.0/*μ*L, respectively (Table 1). A chest radiograph showed a mediastinal shift to the right toward the atelectasis, a reticulonodular pattern on the right lower lobe, and nodular density on the left middle lobe (Fig. 1A). Computed tomography (CT) scans of the lungs revealed partial atelectasis of the lower right lobe owing to mucoid impaction, and bronchiectasis (Fig. 1D and E). There were no visible bacteria upon Gram staining of sputum samples.

**Table 1:** Laboratory data

Hematology		Biochemistry		Serology	
WBC	1100/*μ*L	TP	5.4 g/dL	CRP	27.54 mg/dL
Neut	63.6%	Alb	2.8 g/dL	IgG	412 mg/dL
Lymph	10.3%	AST	12 IU/L	IgA	56 mg/dL
Mono	25.2%	ALT	9 IU/L	IgE	308 IU/mL
Eosino	0.9%	LDH	285 IU/L	*β*‐D‐glucan	89.7 pg/mL
Baso	0%	ALP	211 IU/L	Aspergillus GM	Positive
RBC	227 × 10^4^/*μ*L	BUN	16.4 mg/dL		
Hb	6.9 g/dL	Cre	0.79 mg/dL	Arterial blood gas analysis (5 L mask)	
Ht	21.1%	Na	133 mEq/L	pH	7.457
Plt	10.9 × 10^4^/*μ*L	K	3.4 mEq/L	pCO_2_	35.1 mmHg
CD4	47.1%	Cl	101 mEq/L	pO_2_	79.2 mmHg
CD8	45.5%	BS	260 mg/dL	HCO_3_	24.2 mmol/L
Coagulopathy					
PT‐INR	1.2				
APTT	35.7 sec				

**Figure 2:**
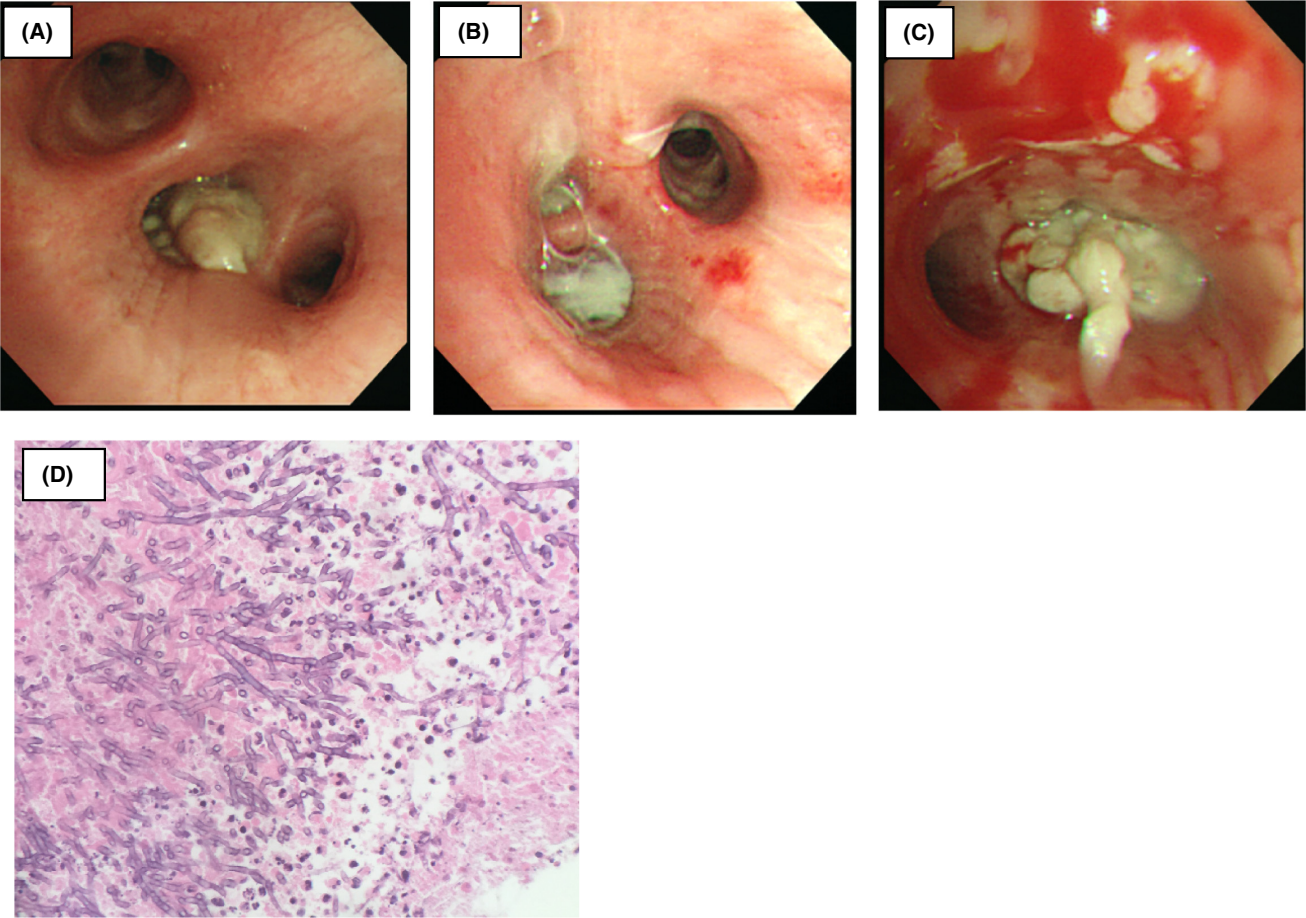
Numerous white mucoid plugs blocked both side of the main and distal bronchi (A; right bronchus intermedius, B; left superior segment, C; right middle lobar branch). Cytology of the sputum showed that there were many fungal filaments of the Y letter type (D; hematoxylin and eosin staining, 400x).

On the night of admission, the patient developed sudden severe respiratory failure (Fig. 1B), which rapidly deteriorated (approximate oxygen saturation, 80%); a 15‐L/min oxygen supply was administered via a mask, and the patient was intubated because of a requirement for mechanical ventilation at the intensive care unit. Empirical antibiotic therapy with parenteral meropenem (3.0 g/day) and liposomal amphotericin B (100 mg/day) was administered. A bronchoscopic examination was performed on the second day of hospitalization (Fig. 2); numerous white mucoid plugs of the upper and lower lobe bronchi were observed, and sputum was removed by suction. In addition, inconsistent with obstructing bronchial aspergillosis, during the bronchoscopy, we observed pseudomembrane formation in the bronchial walls, showing *Aspergillus* invasion of the bronchial mucosa. Sputum cytology showed many branching, septate hyphae; sputum culture revealed the presence of *A. fumigatus* and *A. flavus*. The *A. fumigatus*‐specific immunoglobulin E (IgE) antibody test was positive (16.90 UA/mL). Further investigation revealed a positive type 1 skin reaction to *A. fumigatus* and a total IgE concentration of 308.0 IU/mL; however, the serum antibody test for *Aspergillus* IgG antibodies was negative.

The patient reported an improvement in respiratory discomfort following the removal of the mucoid plugs. On day 8 following hospitalization, the patient was extubated because her breathing improved, indicating that mechanical ventilation was no longer required. A chest radiograph showed that the atelectasis improved (Fig. 1C), and a subsequent chest CT scan showed that the ground‐glass opacity and mucoid impaction were improved. On day 21 following admission, liposomal amphotericin B therapy was switched to itraconazole (200 mg/day); the plan was to treat the patient with antifungal therapy for 16 weeks. Eight weeks following the initiation of RIT treatment, the patient's left cervical and intraabdominal lymphadenopathy was revealed. Although the pneumonia shadow had nearly disappeared, it was estimated that the malignant lymphoma had recurred. Salvage therapy for recurrent lymphoma was initiated, but the patient subsequently died as a result of central nervous system involvement.

## Discussion

Invasive tracheobronchial aspergillosis without parenchymal disease is relatively rare and observed in <10% of IPA cases. Carol et al. 8 reported ulcerative and plaque‐like tracheobronchitis due to infection with aspergillus in 58 AIDS patients. Fernandez‐Ruiz et al. reviewed 148 previously reported cases of aspergillus tracheobronchitis, highlighting that the most commonly reported underlying conditions were solid organ transplantation (44.2%), hematologic malignancy (21.2%), neutropenia (18.7%), and chronic obstructive pulmonary disease (15.4%) 9. ITBA mimicked allergic bronchopulmonary aspergillosis (ABPA) on CT scans and manifested as bilateral bronchial and bronchiolar dilatation, large mucoid impactions, and diffuse lower lobe consolidation caused by postobstructive atelectasis. Characteristic CT findings in angioinvasive aspergillosis consist of nodules surrounded by a halo of ground‐glass attenuation (“halo sign”) or pleura‐based, wedge‐shaped areas of consolidation. Although this case was considered ABPA or obstructive bronchial aspergillosis, we diagnosed the patient with ITBA because of findings inconsistent with ABPA, such as the development of acute respiratory failure, increased CRP levels, the presence of many fungal hyphae but no evidence of eosinophils (allergic mucin) in the sputum, and the absence of serum antibodies against *Aspergillus* IgG. Notably, additional examination with bronchial biopsy would make this diagnosis more reliable. In this case, decreased cell‐mediated immunity, owing to previously administered R‐Hyper CVAD/MA treatment and RIT therapy, in addition to a period of neutropenia, owing to RIT, led to the patient becoming infected with *Aspergillus* and subsequently developing ITBA. Furthermore, the immunosuppressive state contributed to the low levels of specific IgG.

We report a case of the development of ITBA after RIT therapy, with severe immunodeficiency, owing to prior chemotherapy, which we successfully managed using liposomal amphotericin B.

## Authorship

SS, YT: prepared the manuscript and provided the pathology description. HS, SW, and TK: provided the images. EK and ET: assisted with the literature review and edited the final manuscript.

## Conflict of Interest

None declared.
